# People-selectivity, audiovisual integration and heteromodality in the superior temporal sulcus

**DOI:** 10.1016/j.cortex.2013.07.011

**Published:** 2014-01

**Authors:** Rebecca Watson, Marianne Latinus, Ian Charest, Frances Crabbe, Pascal Belin

**Affiliations:** a Maastricht Brain Imaging Center, Faculty of Psychology and Neuroscience, Maastricht University, Maastricht, The Netherlands; b Centre for Cognitive Neuroimaging, Institute of Neuroscience and Psychology, University of Glasgow, Glasgow, UK; c Institut des Neurosciences de La Timone, UMR 7289, CNRS & Université Aix-Marseille, Marseille, France; d Medical Research Council-Cognition and Brain Sciences Unit (MRC-CBU), Cambridge, UK; e International Laboratories for Brain, Music and Sound (BRAMS), Université de Montréal & McGill University, Montreal, Canada

**Keywords:** Audiovisual integration, Superior temporal sulcus, Face sensitivity, Voice sensitivity

## Abstract

The functional role of the superior temporal sulcus (STS) has been implicated in a number of studies, including those investigating face perception, voice perception, and face–voice integration. However, the nature of the STS preference for these ‘social stimuli’ remains unclear, as does the location within the STS for specific types of information processing. The aim of this study was to directly examine properties of the STS in terms of selective response to social stimuli. We used functional magnetic resonance imaging (fMRI) to scan participants whilst they were presented with auditory, visual, or audiovisual stimuli of people or objects, with the intention of localising areas preferring both faces *and* voices (i.e., ‘people-selective’ regions) and audiovisual regions designed to specifically integrate person-related information. Results highlighted a ‘people-selective, heteromodal’ region in the trunk of the right STS which was activated by both faces and voices, and a restricted portion of the right posterior STS (pSTS) with an integrative preference for information from people, as compared to objects. These results point towards the dedicated role of the STS as a ‘social-information processing’ centre.

## 1 Introduction

In the last decade, the human superior temporal sulcus (STS) and surrounding areas have been widely studied (see Hein & Knight, 2008 for a review). The STS is a major sulcal landmark in the temporal lobe, lying between cortices on the surface of the superior temporal gyrus (STG) and middle temporal gyrus (MTG). An extensive region, it can be divided into three distinct sections: the anterior, mid, and posterior STS (aSTS, mid-STS, pSTS). Furthermore, in most individuals, the pSTS divides into two spatially separable terminal ascending branches – the so-called anterior and posterior terminal ascending branches. Thus, the STS can also be anatomically separated into the branch, bifurcation (equivalent to pSTS) and trunk parts (equivalent to mid-STS, aSTS) (Ochiai et al., 2004). There is now a large body of evidence which suggests the STS is a major player in social perception – particularly, the pSTS region. This evidence has been provided from two separate camps of research; the first which has investigated unimodal face and voice processing, and the second which has pointed to the role of the pSTS in multisensory integration of social signals (Allison, Puce, & McCarthy, 2000).

We rely greatly on information gathered from both facial and vocal information when engaging in social interaction. Along with the inferior occipital gyri (IOGs) and lateral fusiform gyrus (FG) [specifically, the fusiform face area (FFA) (Kanwisher, McDermott, & Chun, 1997)] the pSTS has been highlighted as a key component of the human neural system for face perception (Haxby, Hoffman, & Gobbini, 2000). It appears to be particularly involved in processing the more dynamic aspects of faces: when attending to these aspects the magnitude of the response to faces in the FFA is reduced and the response in the pSTS increases (Hoffman & Haxby, 2000). Although perhaps not as strong as for faces, evidence for voice-selective regions, particularly in the STS, is accumulating. Several fMRI studies (e.g., Belin et al., 2000, Ethofer et al., 2009, Grandjean et al., 2005, Linden et al., 2011) have demonstrated the existence of voice-selective neuronal populations: these voice-selective regions of cortex [‘temporal voice areas’ (TVAs)] are organized in several clusters distributed antero-posteriorly along the STG and STS bilaterally, generally with a right-hemispheric preponderance (Belin et al., 2000, Kreifelts et al., 2009). The aSTS and pSTS in particular appear to play an important role in the paralinguistic processing of voices, such as voice identity (Andics et al., 2010, Belin and Zatorre, 2003, Latinus et al., 2011). Thus parts of the pSTS appear to show greater response to social signals compared to non-social control stimuli in both the visual and auditory modalities, although the relative location of face- and voice-sensitive regions in pSTS remains unclear.

Turning away from unimodal face and voice processing, another vital skill for effective social communication is the ability to combine information we receive from multiple sensory modalities into one percept. Converging results point to the role of the pSTS in multisensory integration, particularly in audiovisual processing. The logic of fMRI experiments on audiovisual integration has been to search for brain regions which are significantly involved in the processing of unimodal visual and auditory stimuli, but show an even stronger activation if these inputs are presented together—the so-called ‘supra-additive response’, where the response to the bimodal stimuli is larger than the sum of the unimodal responses. Integration of speech (Calvert et al., 2000, Wright et al., 2003), affective (Ethofer et al., 2006, Kreifelts et al., 2009, Pourtois et al., 2005), and identity (Blank, Anwander, & von Kriegstein, 2011) information from faces and voices have all been found in the pSTS. However, it should also be noted that integration of ‘non-social’ information – such as tools and their corresponding sounds (Beauchamp, Lee, Argall, & Martin, 2004) and letters and speech sounds (van Atteveldt, Formisano, Goebel, & Blomert, 2004) – has also been observed in the pSTS, and thus it is unclear whether this region performs a more ‘general’ integrative role, or shows preferences for particular stimulus categories.

Here we brought together these distinct lines of research by examining properties of the STS in terms of selective response to social stimuli. Normal adult volunteers participated in an ‘audiovisual localiser’ scan during which they were stimulated with auditory, visual, or audiovisual stimuli of people or objects. We proposed, given that face-selective, voice-selective and integrative regions are found within the STS, that in addition to areas preferring both faces *and* voices (i.e., ‘people-selective’ regions) there could also be audiovisual regions that are more sensitive to social stimuli, as compared to information from non-social categories, such as objects.

We found that a restricted portion of the right pSTS was characterised by a conjunction of (1) an ‘integrative’ response, i.e., stronger response to audiovisual stimuli compared to visual and compared to auditory stimuli and (2) ‘people-selectivity’, i.e., preference for social stimuli irrespective of the modality (voice > objects; face > objects). Furthermore, a large region further extending down the trunk of the right STS was observed to be heteromodal: that is, this region was activated by both faces and voices, but did not necessarily show integrative properties.

## 2 Materials and methods

### 2.1 Participants

Forty English-speaking participants (15 males and 25 females; mean age: 25 years ± 5 years) took part in the scan. All had self-reported normal or corrected vision and hearing. The ethical committee from the University of Glasgow approved the study. All volunteers provided informed written consent before, and received payment for, participation.

### 2.2 Stimuli

**Fig. 1:**
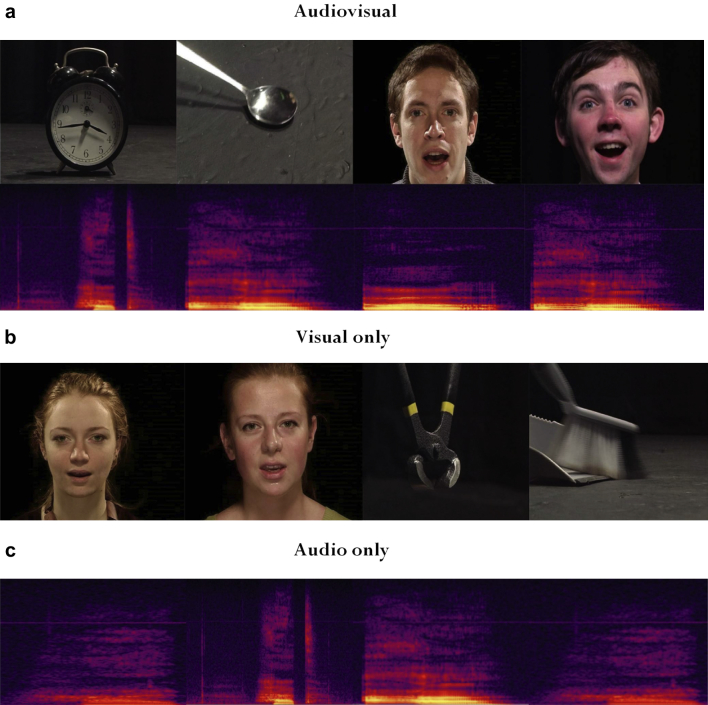
Examples of (a) audiovisual, (b) visual and (c) auditory stimuli. Stimuli for the audiovisual localiser are available at http://vnl.psy.gla.ac.uk/resources.

24 people (12 males and 12 females) were video-recorded producing a variety of vocal expressions, both speech and non-speech (e.g., saying the word ‘had’, humming, yawning). Recordings took place in the television studio at the Learning and Teaching Centre, Glasgow University, and participants were paid at the rate of £6 per hour. The participants were filmed under standard studio lighting conditions (standard tungsten light), and sat directly facing the camera, at a distance so that the whole face was in frame. Videos were recorded with 25 frames per second (40 msec per frame) using a Panasonic DVC Pro AJD 610 camera, fitted with a Fujiform A17 × 7.8 BERM-M28 lens, and transferred and edited using Adobe Premier Elements. Within the video recording, vocalisations were recorded with 16-bit resolution at a sampling frequency of 44,100 Hz. Under the same conditions, 24 moving objects producing sound were also filmed (e.g., a moving toy car, a ball bouncing, a violin being played). The objects were filmed with the intention of recording the canonical view. Videos were edited so that every production of a vocal sound by a participant formed a separate clip, with the clips lasting 2 sec each. The videos of the objects were edited to form separate clips of 2 sec each also. For examples of stimuli, please refer to Fig. 1.

Stimulus clips were combined together in Adobe Premier Elements to form 18 different 16 sec blocks. Thus, each block contained eight different stimuli. These blocks were broadly categorised as:

(1) Faces paired with their corresponding vocal sounds (AV-P)
(2) Objects (visual and audio) (AV-O)
(3) Voices alone (A-P)
(4) Objects (audio only) (A-O)
(5) Faces alone (V-P)
(6) Objects (visual only) (V-O)

Thus, categories 1 and 2 were audiovisual; 3 and 4 were audio only; and 5 and 6 were visual only. There were three different stimulus blocks of each type, each containing different visual/auditory/audio-visual stimuli. A 16-sec null event block comprising silence and a grey screen was also created. Each of the 18 blocks was repeated twice, and the blocks were presented pseudo-randomly: each block was always preceded and followed by a block from a different category (e.g., a block from the ‘Faces alone’ category could never be preceded/followed by any other block from the ‘Faces alone’ category). The null event block was repeated six times, and interspersed randomly within the presentations of the stimulus blocks.

### 2.3 Design and procedure

Stimuli were presented using the Psychtoolbox in Matlab, via electrostatic headphones (NordicNeuroLab, Norway) at a sound pressure level of 80 dB as measured using a Lutron Sl-4010 sound level metre. Before they were scanned, subjects were presented with sound samples to verify that the sound pressure level was comfortable and loud enough considering the scanner noise. Stimuli were presented in one scanning run while blood oxygenation-level dependent (BOLD) signal was measured in the fMRI scanner. Participants were not required to perform an active task; however, they were instructed to pay close attention to the stimuli.

### 2.4 Imaging parameters

Functional images covering the whole brain (slices = 32, field of view = 210 × 210 mm, voxel size = 3 × 3 × 3 mm) were acquired on a 3 T Tim Trio Scanner (Siemens) with a 12-channel head coil, using an echoplanar imaging (EPI) sequence [interleaved, TR = 2 sec, TE = 30 msec, Flip Angle (FA) = 80°]. We acquired 336 EPI volumes for the experiment. The first 4 sec of the functional run consisted of ‘dummy’ gradient and radio frequency pulses to allow for steady state magnetisation during which no stimuli were presented and no fMRI data collected. MRI was performed at the Centre for Cognitive Neuroimaging (CCNi) in Glasgow, UK.

At the end of each fMRI session, high-resolution T1-weighted structural images were collected in 192 axial slices and isotropic voxels (1 mm^3^; field of view: 256 × 256 mm, TR = 1900 msec, TE = 2.92 msec, time to inversion = 900 msec, FA = 9°).

### 2.5 Imaging analysis

SPM8 software (Wellcome Department of Imaging Neuroscience, London, UK; http://www.fil.ion.ucl.ac.uk/spm) was used to pre-process and analyse the imaging data. First the anatomical scan was AC–PC centred, and this correction applied to all EPI volumes.

Functional data were motion corrected using a spatial transformation which realigned all functional volumes to the first volume of the run and subsequently realigned the volumes to the mean volume. The anatomical scan was co-registered to the mean volume and segmented. The anatomical and functional images were then normalised to the Montreal Neurological Institute (MNI) template using the parameters issued from the segmentation keeping the voxel resolution of the original scans (1 × 1 × 1 and 3 × 3 × 3 respectively). Functional images were then smoothed with a Gaussian function (8 × 8 × 8 mm).

EPI time series were analysed using the general linear model as implemented in SPM8. Functional data were analysed in one two-level random-effects design. The first-level, fixed-effects individual participant analysis involved a design matrix containing a separate regressor for each block category (1–6). These regressors contained boxcar functions representing the onset and offset of stimulation blocks convolved with a canonical haemodynamic response function (HRF). To account for residual motion artefacts the realignment parameters were also added as nuisance covariates to the design matrix. Using the modified general linear model parameter estimates for each condition at each voxel were calculated and then used to create contrast images for each category relative to baseline: AV-P > baseline, AV-O > baseline, A-P > baseline, A-O > baseline, V-P > baseline, V-O > baseline. These six contrast images, from each participant, were taken forward into the second-level two factor (modality and category) ANOVA. The order of conditions was: Audiovisual (Person); Audiovisual (Object); Audio only (Person); Audio only (Object); Visual only (Person); Visual only (Object).

Stimulus condition effects were tested with A(P + O) > baseline for sounds, V(P + O) > baseline for images and AV(P + O) > baseline for cross-modal sound-image. These contrasts were thresholded at *p* < .05 (FWE peak voxel corrected) with a minimum cluster size of five contiguous voxels.

The inclusion of non-face and non-vocal stimuli also allowed us to examine selectivity for faces and voices. We identified face-selective and voice-selective regions, firstly with inclusion of audiovisual conditions (i.e., AV-P + V-P > AV-O + V-O for face selective, AV-P + A-P > AV-O + A-O for voice selective), and then with only unimodal conditions included. These contrasts were thresholded at *p* < .05 (FWE correction for cluster size) in conjunction with a peak voxel threshold of *p* < .0001 (uncorrected). In addition, we imposed a minimum cluster size of 10 contiguous voxels.

We then identified ‘people-selective’ regions as those who showed a ‘person-preferring’ response, regardless of the condition, whether this was audiovisual, audio only, or visual only (i.e., AV-P + A-P + V-P > AV-O + A-O + V-O). This contrast was thresholded at *p* < .05 (FWE peak voxel corrected) with a minimum cluster size of 10 contiguous voxels.

#### 2.5.1 Conjunction analyses

We further performed a series of conjunction analyses in SPM8 in order to identify regions meeting a number of functional criteria:

##### 2.5.1.1 Audiovisual integration

We tested for general audiovisual, integrative regions with the conjunction analysis AV(P + O) > V(P + O) ∩ AV(P + O) > A(P + O) [i.e., the ‘max rule’ (Beauchamp, 2005, Love et al., 2011)]. This localised regions which showed a higher response to audiovisual stimuli as compared to both visual only and audio only stimuli.

We then tested for audiovisual regions which were also people selective [AV(P + O) > V(P + O) ∩ AV(P + O) > A(P + O) ∩ (AV-P + A-P + V-P > AV-O + A-O + V-O)].

##### 2.5.1.2 Heteromodal response

We tested for regions that responded to both auditory and visual information (irrespective or their response to audiovisual stimuli) with the conjunction analysis A(P + O) ∩ V(P + O). It is important to note that alongside identifying heteromodal regions, integrative regions could also emerge from this criterion, as there was no criteria/requirement regarding the strength of the AV response.

We then tested for heteromodal regions that were also ‘people selective’ with the conjunction A(P + O) ∩ V(P + O) ∩ (AV-P + A-P + V-P > AV-O + A-O + V-O).

For all conjunction analyses, results were thresholded at *p* < .05 (FWE peak voxel corrected) with a cluster extent threshold of *k* > 5.

## 3 Results

Regions activating more to auditory information (voices and object sounds) than the baseline condition were bilateral auditory cortex, right inferior frontal gyrus (IFG), and bilateral middle frontal gyrus (MFG) (Table 1a). Regions activating more to visual information (silent faces and objects) than the baseline condition were the broad visual cortex, bilateral STG, left medial frontal gyrus, bilateral IFG, right superior frontal gyrus (SFG), the posterior cingulate and the precuneus (Table 1b). Regions activating more to audiovisual stimuli than baseline were bilateral visual and auditory cortex, bilateral IFG and right medial frontal gyrus (Table 1c).

**Table 1:** Stimulus condition effects. Results of independently contrasting unimodal (a and b) and audiovisual (c) conditions against baseline.

Brain regions	Coordinates (mm)			*k*	*t*-statistic
	*x*	*y*	*z*		
**(a) A > baseline**					
STG	−48	−25	7	1846	20.76
STG	51	−22	4	2062	20.14
IFG	39	17	25	112	6.22
MFG	−42	17	25	136	6.11

**(b) V > baseline**					
Middle occipital gyrus (MOG)	45	−70	1	6135	24.21
IFG	42	11	28	650	9.30
Superior parietal lobule	30	−55	49	145	7.74
IFG	−39	11	22	272	7.74
IFG	30	32	−14	47	6.29
SFG	3	59	34	20	5.52
Medial frontal gyrus	−3	53	−14	27	5.50
Posterior cingulate gyrus	0	−52	16	22	5.43
Precuneus	−27	−55	49	15	4.96

**(c) AV > baseline**					
MOG	45	−70	1	8670	22.65
IFG	42	14	25	608	10.38
IFG	−39	11	22	123	7.34
Precentral gyrus	−48	−1	49	48	5.82
Medial frontal gyrus	6	59	4	11	5.55
IFG	27	32	−11	19	5.35
IFG	−39	29	1	13	5.22
Superior parietal lobule	30	−55	49	11	5.03

Contrasts were height thresholded (*t* = 4.51) to display voxels reaching a significance level of *p* < .05 with FWE correction and an additional minimum cluster size of greater than five contiguous voxels. MNI coordinates and *t*-scores are from the peak voxel of a cluster.

Face-selective regions were found in the right STG and left MTG, the right MFG, precuneus and caudate. At a more liberal threshold [*p* < .001 (uncorrected)], the right IFG and right FFA emerged as face-selective regions (see Table 2a and b). Voice-selective regions were found in the bilateral STG/MTG, precuneus and right MFG (Table 2c and d).

**Table 2:** Face and voice-selective regions. Results of independently contrasting faces and voices against object images and non-vocal sounds (a, b and c, d respectively).

Brain regions	Coordinates (mm)			*k*	*t*-statistic
	*x*	*y*	*z*		
**(a) Face-selective regions (including AV information)**					
STG/STS	51	−34	1	867	13.98
MFG	51	2	46	735	9.05
MTG	−60	−16	−5	405	8.12
Precuneus	3	−58	31	249	7.72
IOG	27	−97	−5	45	5.79*

**(b) Face-selective regions (excluding AV information)**					
STG/STS	51	−37	4	820	10.51
MFG	51	−1	46	856	8.86
Precuneus	3	−58	28	197	5.62
STG/STS	−57	−40	7	171	4.88
Caudate	18	−4	16	184	4.56
IOG	42	−82	−11	72	5.38*
FG	42	−46	−17	13	4.20*

**(c) Voice-selective regions (including AV information)**					
STG/STS	51	−34	1	521	12.08
MTG	−60	−10	−8	295	9.25
Precuneus	3	−58	28	99	7.12
MFG	45	20	25	45	5.56

**(d) Voice-selective regions (excluding AV information)**					
STG/STS	57	−19	−5	247	5.03
STG	−60	−10	−8	105	4.12
Precuneus	3	−58	28	33	3.69

Contrasts were height thresholded (*t* = 3.13) to display voxels reaching a significance level of *p* < .0001 combined with an FWE correction of *p* < .05 for cluster size. MNI coordinates and *t*-scores are from the peak voxel of a cluster.

∗Contrasts were significant at a peak voxel threshold of *p* < .0001 (uncorrected), with no cluster thresholding.

**Fig. 2:**
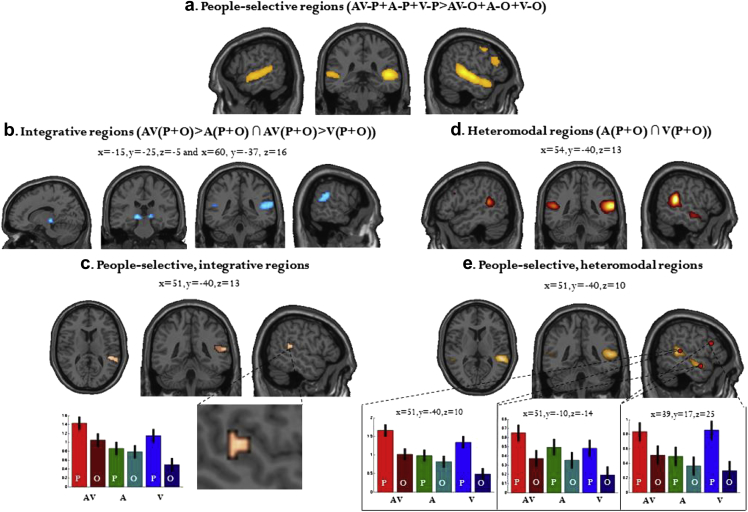
People-selectivity, audiovisual integration and heteromodality: (a) ‘People-selective’ regions, defined by a contrast of AV-P + A-P + V-P > AV-O + A-O + V-O*; (b) Integrative audiovisual regions, defined by a contrast of AV(P + O) > A(P + O) ∩ AV(P + O) > V(P + O); (c) Conjunction of a and b: Integrative, people-selective regions; (d) Heteromodal regions; (e) Conjunction of a and d: Heteromodal, people-selective regions. Contrasts were height thresholded (*t* = 4.52) to display voxels reaching a significance level of *p* < .05 with FWE correction and an additional minimum cluster size of greater than five contiguous voxels. MNI coordinates and *t*-scores are from the peak voxel of a cluster. *AV = audiovisual; V = visual; A = auditory; P = people; O = objects.

Regions which showed a greater response to people-specific information as compared to object-specific information (regardless of the modality) included the bilateral STG, bilateral IFG, the right precuneus, and right hippocampus (Table 3a/Fig. 2a).

**Table 3:** People-selective regions. Results of independently contrasting people-related information against object related information, regardless of condition.

Brain regions	Coordinates (mm)			*k*	*t*-statistic
	*x*	*y*	*z*		
**(a) ‘People-selective’ regions**					
STG/STS	51	−34	1	710	15.01
STG	−60	−16	−5	324	9.25
IFG	42	20	25	406	8.85
Precuneus	3	−58	28	187	8.83
Hippocampus	21	−7	−14	25	6.39
IFG	−39	14	22	11	4.96

Contrasts were height thresholded (*t* = 4.51) to display voxels reaching a significance level of *p* < .05 (FWE corrected for multiple comparisons). MNI coordinates and *t*-scores are from the peak voxel of a cluster.

### 3.1 Conjunction analyses

#### 3.1.1 Audiovisual, integrative regions

**Fig. 3:**
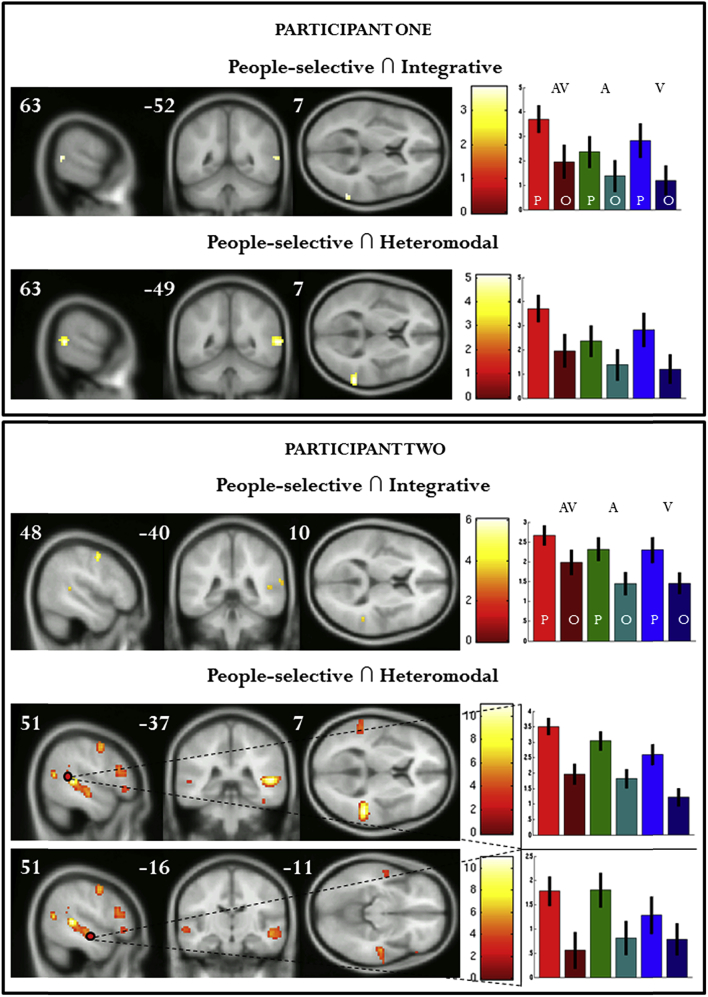
Results from individual participants: people-selective, integrative regions and people-selective, heteromodal regions. For descriptive purposes, contrasts are height thresholded (*t* = 3.12) to display voxels reaching a significance level of *p* < .001 (uncorrected). MNI coordinates and *t*-scores are from global and local (Participant 2) maxima of STS cluster.

Audiovisual integrative regions (regardless of stimulus category), i.e., following the ‘max rule’ [AV(P + O) > A(P + O) ∩ AV(P + O) > V(P + O)] were found in the bilateral thalamus and bilateral STG/STS (Table 4a/Fig. 2b). An integrative, people-selective region, i.e., a region following both the max rule and showing an average greater response to people than object in audition (voice > object) and vision (face > object) was observed in the right STG/pSTS (Table 4b/Fig. 2c). This region can also be seen at the level of individual participants in Fig. 3.

**Table 4:** Results of conjunction analyses: (a) Integrative audiovisual regions (AV > A ∩ AV > V); (b) Integrative, people-selective regions; (c) Heteromodal regions (Auditory > Baseline ∩ Visual > Baseline); (d) Heteromodal, people-selective regions.

Brain regions	Coordinates (mm)			*k*	*t*-statistic
	*x*	*y*	*z*		
**(a) Integrative regions (max rule: AV > A ∩ AV > V)**					
Thalamus	−15	−25	−5	21	7.04
STG/STS	60	−37	16	108	6.18
Thalamus	15	−25	−5	10	5.83
STG	−51	−46	13	14	5.36

**(b) People-selective integrative regions**					
STG/STS	51	−40	13	52	5.97

**(c) Heteromodal regions (A ∩ V)**					
STG/STS	54	−40	13	575	11.10
STG/STS	−54	−46	13	183	8.51
IFG	39	17	25	109	6.15
IFG	−42	14	25	95	6.08
STG	36	2	−20	16	5.56

**(d) People-selective heteromodal regions**					
STG/STS	51	−40	10	325	10.50
IFG	39	17	25	108	6.22
IFG	−39	14	22	11	4.96

Contrasts were height thresholded (*t* = 4.52) to display voxels reaching a significance level of *p* < .05 with FWE correction and an additional minimum cluster size of greater than five contiguous voxels. MNI coordinates and *t*-scores are from the peak voxel of a cluster.

As an additional test of our results, we defined integrative regions using one half of the data, which highlighted clusters in the right and left posterior superior temporal gyrus/sulcus (pSTG/STS; see Table 5a). Within each of these clusters, we then tested to see whether people-selectivity – as defined using the other half of the data – was significant. Within the left pSTS, this contrast was not significant (*t* = −.46, *p* = .675); however, within the right pSTS this elicited a significant effect (*t* = 3.06, *p* < .002). This appears to confirm our initial finding that this particular cluster in the right pSTS is both people-selective and integrative.

**Table 5:** Integrative and heteromodal regions as defined using one half of the data: (a) Integrative, audiovisual regions (AV > A ∩ AV > V); (b) Heteromodal regions (A ∩ V).

Brain regions	Coordinates (mm)			*k*	*t*-statistic
	*x*	*y*	*z*		
**(a) ‘Integrative’ regions (AV > A ∩ AV > V)**					
STG/STS	51	−40	10	135	6.68
STG/STS	−51	−46	13	22	5.76

**(b) ‘Heteromodal’ regions (A ∩ V)**					
STG/STS	54	−40	13	119	7.00
STS	−54	−46	13	13	5.45

Contrasts were height thresholded (*t* = 4.57) to display voxels reaching a significance level of *p* < .05 with FWE correction and an additional minimum cluster size of greater than five contiguous voxels. MNI coordinates and *t*-scores are from the peak voxel of a cluster.

#### 3.1.2 Heteromodal regions

Regions which responded to both visual and auditory information, as compared to baseline, consisted of the bilateral STG, and bilateral inferior frontal gyri (Table 4c/Fig. 2d). Note that whereas the ‘heteromodality’ criterion does not make any assumption on what should be the response to the AV condition, a large part of the right pSTS also followed the ‘max rule’. People-selective heteromodal regions, i.e., regions that responded significantly to both auditory and visual stimuli and that preferred social stimuli in both modalities, extended anteriorly to a large part of the STG/STS, and also activated the bilateral IFG (Table 4d/Fig. 2e). These regions can also be seen at the level of individual participants in Fig. 3.

Similarly to the previous analysis, we defined heteromodal regions using one half of the data, which highlighted clusters in the right and left pSTG/STS; see Table 5b. Within each of these clusters, we then tested to see whether people-selectivity – as defined using the other half of the data – was significant. Within the left pSTS, this contrast was not significant (*t* = −.15, *p* = .56); however, within the right pSTS this elicited a significant effect (*t* = 2.96, *p* < .002).

## 4 Discussion

The aim of this study was to examine the neural correlates of people-selectivity (i.e., regions that preferred face and voice information, regardless of condition), audiovisual integration (i.e., a significantly stronger response to audiovisual as compared to unimodal stimuli), and ‘heteromodality’ (i.e., a significant response to both vision and audition), specifically within the pSTS. Participants were scanned during an ‘audiovisual localiser’ during which they passively viewed a series of audiovisual, visual and auditory stimuli of either people or objects; responses to each specific condition were compared and contrasted. Using a single dataset and ecological stimuli – dynamic movies of faces and voices – our results not only confirm the multisensory nature of the pSTS, but also that areas of this structure selectively process person-related information irrespective of the sensory modality.

### 4.1 Face-selectivity, voice-selectivity and people-selectivity in the STS

We firstly examined voice- and face-selectivity in our participants by contrasting the response to voices as compared to non-vocal sounds, and faces as compared to visual representations of objects, respectively.

When we contrasted the response to auditory information against baseline, the broad auditory cortex was highlighted bilaterally. A voice-selective response was confined to the upper banks of the bilateral STS; regions that appear to correspond with the ‘TVAs’ identified by Belin et al. (2000) and Belin, Fecteau, and Bédard (2004). Maximum voice-selectivity was found in the mid-STS, a result which has been found in a number of other studies (e.g., Belin et al., 2002, Belin et al., 2000, Kreifelts et al., 2009). The ‘voice-selective’ regions of the STS tend to show a greater response to vocal sounds than to non-vocal sounds from natural sources, or acoustical controls such as scrambled voices or amplitude-modulated noise. This response is also observed for vocal sounds of non-linguistic content (Belin et al., 2011, Belin et al., 2002), highlighting that these regions process more than just the speech content of voice. In a voice recognition study, von Kriegstein and Giraud (2004) delineated three distinct areas along the right STS involved in different aspects of voice processing: whereas the mid-anterior STS carries out a spectral analysis of voices, more posterior and anterior areas emphasise more paralinguistic voice processing – for example, identity. We also identified the right precuneus as a voice-selective region in this experiment. Although perhaps less commonly found than the TVA, activation of the precuneus has been apparent in a number of studies investigating voice perception (e.g., von Kriegstein et al., 2003, Sokhi et al., 2005).

The visual versus baseline contrast showed activation maps covering most of the visual ventral stream, including early visual cortex (V1:3), V4, V5/MT, the fusiform and parahippocampal gyri and an extensive part of the human inferior temporal (IT) gyrus. This is consistent with the vast majority of research studying visual responsiveness. Face-selectivity was found in a network of regions, including the extensive right STS, left pSTS to mid-STS, the MFG, precuneus and caudate – all regions which have been associated with either the core or extended face-processing system (e.g., Andrews et al., 2010, Haxby et al., 2000, Rossion et al., 2003). Notably, at the set-threshold for the group-level analysis, the commonly found FFAs did not emerge, although these regions – along with the bilateral occipital face areas (OFAs) – did appear for a number of individual participants, as well as at the group level at an uncorrected cluster threshold. Instead, the strongest response appeared to be in the STG/STS – particularly, the right pSTS. In our experiment, we used only dynamic faces, in an attempt to maximise the ecological validity of our stimuli. The pSTS is known to be involved in the representation of the dynamic properties of faces (Allison et al., 2000; Haxby et al., 2000, Haxby et al., 2002) such as mouth, eye and head movements (Lee et al., 2010) and facial expressions (Phillips et al., 1997): although it does respond to pictures of static faces (Hoffman and Haxby, 2000, Kanwisher et al., 1997), it shows a response of significantly greater magnitude (up to three times) to dynamic as compared to static faces (Pitcher, Dilks, Saxe, Triantafyllou, & Kanwisher, 2011). Thus, it could be that continuously presenting only moving faces heightened the response in the pSTS and attenuated the response in the FFA.

We further generalized this approach to all conditions and identified ‘people-selective’ regions in our group of participants as those that responded to social stimuli in all conditions, whether this was audiovisual, audio only or visual only. Such regions were found bilaterally in the pSTS to mid-STS, in addition to the right aSTS, the IFG, hippocampus and precuneus. In a pioneering study, Kreifelts et al. (2009) examined voice-selectivity, face-selectivity and integration of affective information within the STS. They found, using fMRI, that the neural representations of the audiovisual integration of non-verbal emotional signals, voice sensitivity and face sensitivity were located in different parts of the STS with maximum voice sensitivity in the trunk section and maximum face sensitivity in the posterior terminal ascending branch. These authors did not observe the large overlap as was seen in our study, and we can only speculate as to some of the possible reasons. We predict the large response of the STG was in part due to contrasting dynamic audiovisual presentations of people against audiovisual presentations of objects, plus unimodal face and voice information – thus, these would have activated the portions of the STG/STS responsive to audiovisual information, in addition to those responsive to dynamic face information and voice-selective regions. In the study by Kreifelts, face and voice-selectivity were examined using separate localisers, which simply contrasted the response to different sets of unimodal stimuli. What is more, in their face-localiser, the authors only used static faces. Although static faces can also activate the STS (Haxby et al., 2000, Kanwisher et al., 1997) dynamic faces are known to evoke a more pronounced response in this region.

In summary, we find that in this experiment, a large part of the STS – extending from pSTS to aSTS – was overall ‘people selective’: this is striking, considering that previous research has localised face-selectivity and voice-selectivity in different, mostly non-overlapping portions of this region, specifically the pSTS and mid-STS to aSTS, respectively.

### 4.2 Face–voice integration and the STS

We used a conjunction analysis and the classical ‘max criterion’ to define integrative, audiovisual regions in our study. This analysis highlighted the bilateral thalami and the bilateral pSTS as regions responding more to audiovisual information as compared to both visual information and audio information alone.

Both the thalamus and the pSTS are well described as playing a role in multimodal processing. There is now converging evidence that not only sensory non-specific, but also sensory specific, thalamic nuclei may integrate different sensory stimuli and further influence cortical multisensory processing by means of thalamo-cortical feed-forward connections. Some studies provide evidence of thalamic influence on multisensory information processes in rats (Komura, Tamura, Uwano, Nishijo, & Ono, 2005) and humans (Baier, Kleinschmidt, & Müller, 2006) and others link modulations of neuronal activity in subcortical structures with behavioural consequences like audiovisual speech processing (Bushara, Grafman, & Hallett, 2001) and multisensory attention tasks (Vohn et al., 2007). Kreifelts, Ethofer, Grodd, Erb, and Wildgruber (2007) also reported in humans an enhanced classification accuracy of audiovisual emotional stimuli (relative to unimodal presentation) and linked this increase in perceptual performance to enhanced fMRI-signals in multisensory convergence zones, including the thalamus.

The upper bank of the STS has also emerged as a crucial integrative area, particular the pSTS. This region is known to have bidirectional connections with unisensory auditory and visual cortices (Cusick, 1997, Padberg et al., 2003) and to contain around 23% of multisensory neurons (Barraclough, Xiao, Baker, Oram, & Perrett, 2005). Ghazanfar, Maier, Hoffman, and Logothetis (2005) showed that the STS was involved in speech processing when monkeys observed dynamic faces and voices of other monkeys. Consistent with findings from animals, the human pSTS also becomes active when processing audiovisual speech information (Calvert, 2001), in addition to presentations of tools and their corresponding sounds (Beauchamp et al., 2004), letters and speech sounds (van Atteveldt et al., 2004), and faces and voices (Beauchamp et al., 2004; reviewed in Hein & Knight, 2008). Recently – and also using the max criterion – Szycik, Jansma, and Münte (2009) found the bilateral STS to be involved in face–voice integration. Crucially, this was observed using markedly different stimuli to ours – firstly, they presented a static face in their unimodal condition and secondly, they added white noise to their auditory and audiovisual stimuli. The fact that the activation of this region is preserved across stimulus types and sets underlines its importance in the integration of faces and voices. Previously, the hippocampus has also been implicated as key region in the integration of face and voice information (Joassin et al., 2011). At the set-threshold, this region did not emerge: however, as in a recent study by Love et al. (2011), the left hippocampus did emerge at less conservative, uncorrected significance level. This lends further support to the importance of this region; albeit, in a more minor role within this context.

Our conjunction of people-selective and integrative responses highlighted a cluster in the right pSTS, which was more responsive to people-related information – whether this was faces and voices, faces only or voices only. In addition, this region showed a significant preference for audiovisual information, as compared to both audio only and visual only information. Interestingly, this analysis removed the activation previously seen in the thalamus and the left pSTS, suggesting that these regions may be either more ‘general’ – or even, ‘object-selective’ – integrative regions. The right pSTS has been found in previous studies examining audiovisual integration (e.g., Ethofer et al., 2006, Hagan et al., 2009, Kreifelts et al., 2010, Love et al., 2011, Werner and Noppeney, 2010; also reviewed in Calvert, 2001) but crucially, these have generally compared audiovisual to unimodal responses within independent stimulus sets, without contrasting activation to different stimulus categories. To our knowledge, this is the first study that directly looks at person-selectivity of audiovisual integrative regions and we therefore propose that the right pSTS could have a crucial role in combining ‘socially-relevant’ information across modalities.

### 4.3 ‘Heteromodality’ and the STS

Further, we examined responses across modalities: ‘heteromodal’ regions were defined as those that simply responded significantly to both audio and visual information as compared to baseline, irrespective of what their response to the AV condition was. Thus, along with potentially highlighting regions which integrated face and voice information (i.e., showed a significantly stronger response to audiovisual information), this criteria was also able to identify regions which responded to both faces and voices, but did not necessarily integrate this information. This analysis isolated regions in the right pSTS to mid-STS, left pSTS, bilateral IFG and putamen. The bilateral pSTS proved to be an audiovisual, integrative region, overlapping with the regions found in our previous analysis. However, activation continuing down the trunk region of the STS appeared to be genuinely heteromodal: the response to audiovisual information that was not significantly more than either audio or visual presentation, but the auditory and visual responses to the unimodal stimuli were significantly greater than baseline.

When we looked specifically at people-selective portions of these regions, activation followed the line of the posterior to mid-STS. The peak of activation, in the pSTS, again overlapped with people-selective integrative regions. Kreifelts et al. (2010) also observed a sensitivity to voices as well as faces in the right pSTS, which they suggest might be conceived as an essential characteristic of the neural structures subserving the audiovisual integration of human communicative signals. However, they also point out that in their study, given the differences in control stimuli for the separate voice and face-sensitivity experiments, they refrain from any direct comparisons between the two qualities.

Outwith the STS, in the IFG, there was an equal response to both face–voice combinations and faces alone, but a lesser response to voices alone. Interestingly, this ‘heteromodal’ analysis highlighted a multitude of regions that did not emerge using our integrative criterion. We propose that the ‘heteromodality’ criterion, which does not make any assumption on what the response to combined stimuli should be but simply requires a response in both modalities, should not be used as an integrative criterion but could act as an interesting *complement* to the typical analyses used when defining audiovisual regions, especially as some of these defining statistical criteria are recognised as being particularly stringent (Beauchamp, 2005, Love et al., 2011).

### 4.4 People-selectivity and the right hemisphere

In our study we found a strong right-hemispheric response to people-selective information. Although we found an initial people-selective response in both right and left hemispheres, conjunction analyses show lateralised integrative and heteromodal effects in the right hemisphere, specifically the right pSTS to mid-STS, and not in the left hemisphere. Given previous findings on face- and voice-selectivity, this dominance is perhaps unsurprising.

Although studies on face perception have reported face-selective regions in the fusiform gyri of both the left and right cerebral hemispheres, fusiform activations for faces are often found to be greater in the right than in the left (De Renzi et al., 1994, Kanwisher et al., 1997, Le Grand et al., 2003; McCarthy, Puce, Gore, & Allison, 1997), and previous psychophysical investigations with split brain patients also suggest lateral asymmetry in face processing and encoding (Gazzaniga and Smylie, 1983, Miller et al., 2002). In a recent study (Meng, Cherian, Singal, & Sinha, 2012), the authors found that face-selectivity persisted in the right hemisphere even after activity on the left had returned to baseline.

Similarly, studies which have examined voice-selectivity – although smaller in number – also suggest a preference of the right hemisphere. For example, in Belin et al. (2000), the authors observed that averaged in a group of subjects, voice-sensitive activity appeared stronger in the right hemisphere. It appears this asymmetry may be particularly specific to the non-linguistic aspects of voices. In one functional magnetic resonance imaging (fMRI) study (von Kriegstein et al., 2003), it was shown that a task targeting on the speaker's voice (in comparison to a task focussing on verbal content) leads to a response in the right anterior temporal sulcus of the listener. In further study by Belin et al. (2002), it was shown that temporal lobe areas in both hemispheres responded more strongly to human voices than to other sounds (e.g., bells, dog barks, machine sounds) but that, again, it was the right aSTS that responded significantly stronger to non-speech vocalisations than to scrambled versions of the same stimuli. In our experiment, we found bilateral face and voice-selective responses – however, for both of these effects the strongest activation was in the right hemisphere. Given the fact that the linguistic content of our stimuli were kept to a minimum, and that participants passively viewed and heard the visual and auditory information, this right dominance could possibly be expected.

We further identified both integrative and heteromodal regions bilaterally, in the STS and the thalamus (for the former analysis only). However, it was only in the right hemispheres that these effects showed a heightened preference for face and voice information. This extends on the multitude of research that suggests that there is right-hemispheric functional asymmetry in response to social information. Indeed, the right hemisphere shows a preference for not only faces and voices, both also other socially-relevant information such as biological human motion (Beauchamp et al., 2003, Peuskens et al., 2005) and sex pheromones (Savic et al., 2001, Savic et al., 2005). For all of these functions, stronger involvement of the right hemisphere in coding some aspects of person perception seems to be the rule, whereas involvement of the left hemisphere appears to sometimes be a shared role, and only exceptionally a main role. However, the reason to why this ‘social asymmetry’ exists in the first place still remains a relatively open question [see Brancucci, Lucci, Mazzatenta, and Tommasi (2009) for a review]. Additionally, whether the right hemisphere also prefers to integrate these other types of ‘people-selective’ information will only be answered with further investigation.

## 5 Conclusion

Our results build on previous research suggesting that the STS is a ‘social-information processing’ region, by clearly delineating ‘people-selective’ regions that respond discerningly to both face and voice information, across modalities. Furthermore, this study also provides the first evidence of a ‘*people-selective*’ *integrative* region in the right pSTS. Future directions could involve exploring selectivity for other types of socially-relevant information in the STS, inter-individual variability of STS functionality, and further investigating the nature of neuronal populations in ‘people-selective’ STS regions.
